# Role of circRNA circ_0000080 in myocardial hypoxia injury

**DOI:** 10.1080/21655979.2022.2066752

**Published:** 2022-04-27

**Authors:** Bo Wang, Yuyang Zhang, Shunmiao Fang, Hui Wang

**Affiliations:** Department of Cardiology, Xi’an City, Shaanxi Province, China

**Keywords:** Circ_0000080, miR-367-5p, *COX2*, myocardial hypoxia injury

## Abstract

This study aimed to investigate the potential role of circRNA circ_0000080 in myocardial hypoxia injury and the underlying mechanisms. Patients with myocardial hypoxia injury who were admitted to Xi’an No. 1 Hospital, China, were included in this study. The expression levels of circ_0000080, miR-367-5p, and *COX2* were analyzed by real-time quantitative PCR (RT-qPCR); cell viability was measured by cell counting kit-8 (CCK-8) assay; and apoptosis was detected by flow cytometry. In addition, the release of cytokines was determined by Enzyme-linked immunosorbent assay (ELISA), and the binding sites between miR-367-5p and circ_0000080/*COX2* were predicted by bioinformatics analysis and confirmed by dual-luciferase reporter and RNA pull-down assays. circ_0000080 was upregulated in patients with MI and in H9c2 cells treated with H_2_O_2_ and hypoxia/reoxygenation (H/R). Silencing circ_0000080 reduced the H/R-mediated apoptosis of cardiomyocytes and secretion of pro-inflammatory cytokines. Moreover, circ_0000080 functioned as an miR-367-5p sponge to regulate the expression of *COX2*. Downregulated miR-367-5p or overexpressed *COX2* degraded cellular functions of cardiomyocytes. circ_0000080 knockdown alleviated myocardial hypoxia injury through the miR-367-5p/*COX2* axis.

## Graphical abstract

**Figure:**
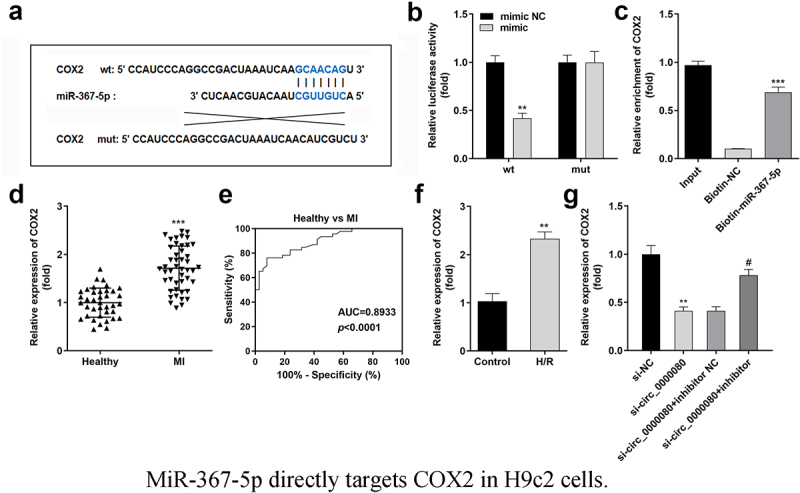

## Highlights

- circ_0000080 is upregulated in patients with MI and in H/R-treated cells.
- MiR-367-5p is a target of circ_0000080.
- MiR-367-5p knockdown reverses the effect of circ_0000080.

## Introduction

Myocardial infarction (MI) is an ischemic cardiomyopathy with high morbidity and mortality, with high global prevalence [1,2]. Myocardial apoptosis induced by oxidative stress can be caused by the sudden and persistent interruption of cardiac blood supply during MI, superoxide overload, and disruption of calcium homeostasis after reperfusion therapy [3,4]. Following apoptosis, myocardial cells are replaced by fibroblasts, leading to systolic dysfunction of the left ventricle and eventually the induction of heart failure [5]. Therefore, uncovering the molecular mechanism underlying myocardial cells is of great importance in the treatment of MI.

Compared to traditional linear RNA, circRNA, with a closed circular structure, is more stable [6,7]. Circular RNAs play critical roles in physiological and pathological processes, and they are involved in the occurrence and development of many diseases, such as cardiovascular diseases [8,9]. Researchers have highlighted the role of circRNA in MI and suggested that the regulation of circFndc3b expression may promote the recovery and remodeling of cardiac function after MI [10]. circMAP3K5 is a major regulator of TET2-mediated SMC differentiation in intimal hyperplasia, as observed in restenosis and atherosclerosis [11]. By inhibiting miR-26b-5p and miR-140-3p, circRNA_000203 may increase GATA4 level and exacerbate cardiac hypertrophy [12]. However, the role of circ_0000080 in cardiomyocyte apoptosis remains unclear.

MicroRNAs are a class of non-coding single-stranded RNA, approximately 21–23 nucleotides long [13,14]. MiR-367-5p is related to cell growth and proliferation; it participates in the occurrence and development of gastric cancer, mandibular protrusion, and other related diseases, and it is important for their diagnosis and treatment [15,16]. To date, the role and mechanism of miR-367-5p in MI remain elusive.

Here, we aimed to reveal the protective effect of circ_0000080 on oxidative stress injury in myocardial cells induced by hypoxia/reoxygenation. We try to elucidate the effect of circ_0000080/miR-367-5p/*COX2* axis on myocardial viability, apoptosis, and inflammation. Overall, this study provides an important theoretical basis for the development of therapeutic targets for treating MI.

## Methods

### Patients and clinical specimen

A total of 38 healthy heart tissues and 45 heart tissues from patients with MI were obtained. The human heart tissue samples were obtained from patients with their consent and their use for research was approved by the ethical review board of Xi’an No. 1 Hospital (ethics number: 2021JM-587).

### Cell culture

Rat embryonic cardiomyocyte H9c2 was purchased from Shanghai Cell Bank of Chinese Academy of Sciences (Shanghai, China). The cells were cultured in Dulbecco’s modified Eagle medium (DMEM) in an incubator at 37°C, with saturated humidity and 5% CO_2_.

### Construction of the myocardial ischemia-reperfusion injury cell model

When the growth density of H9C_2_ cells exceeded 90%, the supernatant of the culture medium was replaced by DMEM without fetal bovine serum. Thereafter, H9c2 cells were placed in an incubator (5% CO_2_ and 95% N_2_) for 4 h, and then cultured in DMEM containing 10% glycerol (5% CO_2_ and 95% O_2_) for 3 h, followed by 24 h of oxygen.

### Cell transfection

Cardiomyocytes were seeded in a 6-well plate. According to the operating instructions, transfection was performed when the degree of cell fusion exceeded 60%. Briefly, Lipofectamine 2000 and plasmid were diluted in Opti-MEM, respectively. After 5 min, the diluted Lipofectamine 2000 and plasmid were mixed evenly. The transfection complex was then added to the 6-well plate and replaced with fresh medium after 4–6 h of transfection. The transfection plasmid used in this study was constructed by Tsingke Biotechnology (Guangzhou, China). SiRNA was transfected with Lipofectamine 2000. The sequences of siRNAs were as follows: si-NC: 5’-AGTGGGTCTACGGCGATA-3’, si-circ_0000080 1#: 5’-GTTTGGAGGAACTCAACCCTA-3’, and si-circ_0000080 2#: 5’-TTTGGAGGAACTCAACCCTAT-3’.

### Real-time quantitative PCR (qRT-PCR)

For fluorescent probe PCR, the following components were added to a 20 μL PCR mixture (catalog No. 4,369,016; ABI, USA): 10 μL TaqMan Gene Expression PCR Master Mix, 1 μL template DNA, 1 μL 20× Prime Probe mixture, 8 μL DNase-free water, and 9.4 μL DNase-free water. The following cycling conditions were used for PCR: 95°C 10 min; 95°C 15s, 60°C 30s, 68°C 30s, 40 cycles. Using the internal reference gene, *GAPDH*, as a reference, the relative content of the target sample gene was calculated. Commercial TaqMan primers for qPCR were purchased from Life Technologies, USA. The primers were: circ_0000080 F: 5’-GAGGAACACTCCATATAATTGGTGA-3’; R: 5’-TCTGTTTTTCTTTGAAGGGCTACCT-3’, miR-367-5p F: 5’-TGCGGACTGTTGCTAATATG-3’; R: 5’-CCAGTGCAGGGTCCGAGGT-3’, COX2 F: 5’-TTCAAATGAGATTGTGGGAAAAT-3’; R: 5’-AGATCATCTCTGCCTGAGTATCTT-3’.

### Cell counting kit-8 (CCK-8) assay

After H9c2 cells were transfected as indicated and treated with hypoxia/reoxygenation (H/R), 10 μL CCK-8 (Catalog No. C0037; Beyotime, Shanghai, China) was added to each well. After 2 h, the absorbance of each sample was measured at 450 nm according to the kit instruction.

### Apoptosis analysis

Flow cytometry was performed according to a previous study [17]. After H9c2 cells were transfected as indicated and treated with H/R, an (annexin V + PI) apoptosis detection kit (Catalog No. C1062L; Beyotime) was used for quantitative analysis by flow cytometry.

### Western blot

Cells were lysed using RIPA buffer. After detecting protein concentration using BCA kit (Catalog No. P0010; Beyotime), equal protein was added and run on 10% SDS-PAGE. Then the protein was transferring to PVDF membranes, followed by blocked with 5% skim milk for 1 h. The membranes were incubated with primary antibodies (anti-Bcl-2: catalog No. ab32124; dilution 1:1000, anti-Bax: catalog No. ab32503; dilution 1:5000, anti-GAPDH: catalog No. ab9485; dilution 1:2500) overnight at 4°C, and incubated with secondary antibody [Goat Anti-Rabbit IgG H&L (HRP): catalog No. ab6721; dilution 1:3000] at room temperature for 1 h. The bands were visualized using ECL plus (Catalog No. P0018M; Beyotime). The original bands were provided in supplemental material 1.

### Enzyme-linked immunosorbent assay (ELISA)

After H9c2 cells were transfected as indicated and treated with H/R, the cell culture supernatant was collected and the concentrations of IL-6, TNF-α, and IL-1β were detected according to the instructions of commercial kits (IL-6 catalog No. 4355; TNF-α catalog No. 920; IL-1β catalog No. 3748; MEIMIAN; Jiangsu, China). ELISA was performed as previous described [18].

### Dual luciferase reporter gene experiment

Target genes were predicted by Starbase v3.0. PMIR-circ_0000080-wt was formed by cloning circ_0000080 cDNA into the pMIR-Vector; pMIR-circ_0000080-mut was inserted by the mutant circ_0000080. The cloned sequences were provided in supplemental material 2. Similarly, pMIR-*COX2*-wt or pMIR-*COX2*-mut was inserted by *COX2* cDNA or mutant *COX2*. When the growth density of H9C_2_ cells exceeded 90%, cells were cultured and co-transfected with pMIR-circ_0000080-wt or pMIR-*COX2*-wt, pMIR-circ_0000080-mut or pMIR-*COX2*-mut, miR-367-5p mimics, and miR-NC, respectively. After 48 h, luciferase was detected with the dual-luciferase reporter gene assay system (catalog No. TM040; Promega, USA) as instructed.

### RNA pull-down assay

The interaction between circ_0000080 and miR-367-5p or the interaction between miR-367-5p and *COX2* in cells was determined by an RNA pull-down assay [17]. Biotin labeled miR-367-5p and biotin-NC were transfected into cells for 72 h. The transfected cells were lysed using cell lysis buffer. Partial split product was used as the input group, and the others were incubated with M-280 streptavidin magnetic beads (catalog No. 11206D; Sigma-Aldrich, USA) at 4°C for 3 h. After washing in low-salt buffer and high-salt buffer, the expression of circ_0000080 and COX2 was measured using qRT-PCR.

### Statistical analysis

SPSS 25.0 statistical software was used for data analysis, and the data are expressed as mean ± SEM. One-way ANOVA was used for comparison among multiple groups, and the LSD test was used for pairwise comparison. P < 0.05 was considered statistically significant.

## Results

In this study, we sought to explore the expression of circ_0000080 in MI and its role in H/R-treated H9c2 cells. We analyzed the effects of circ_0000080 on cell viability, apoptosis, and inflammation. The goal of this study is to provides an important theoretical basis for the development of therapeutic targets for treating MI.

### Circ_0000080 is upregulated in MI and H/R-treated H9c2 cells

**Figure 1.**
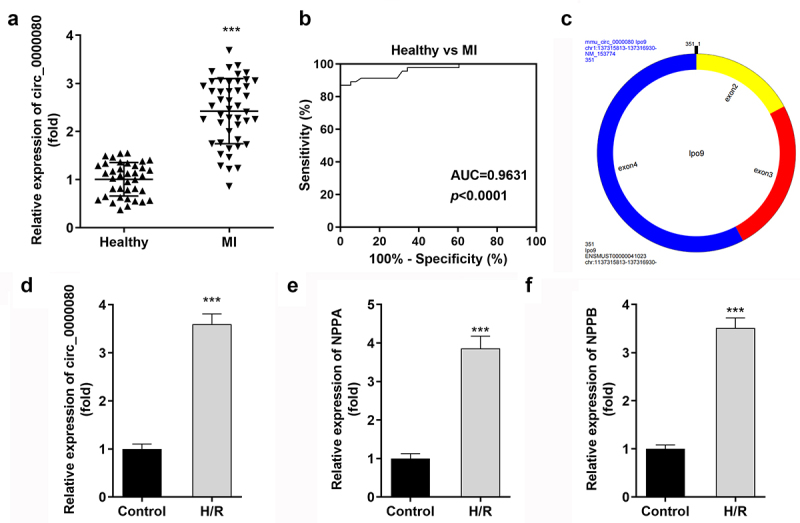
Circ_0000080 is upregulated in patients with MI and in H/R-treated cells. (a) circ_0000080 was upregulated in patients with MI. (b) ROC analysis for the distinction between patients with MI and healthy controls. (c) The information of circ_0000080. (d) The circ_0000080 level was analyzed by qRT-PCR after H/R treatment (n = 3). (e) Detection of NPPA expression in H/R cells (n = 3). (f) Detection of NPPA expression in H/R cells (n = 3). **p < 0.01, ***p < 0.001.

This experiment was conducted to detect the expression level of circ_0000080 between MI patients and healthy individuals (controls) to understand the role of circ_0000080 in the heart. The qRT-PCR results revealed that the expression level of circ_0000080 was significantly elevated in MI patients than in healthy individuals (Figure 1(a)). The corresponding AUC value of circ_0000080 for distinguishing between MI patients and healthy controls was 0.9631 (Figure 1(b)). The information of circ_0000080 was shown in Figure 1(c). Hypoxia/reoxygenation (H/R) treatment increased the circ_0000080 level in H9c2 cells (Figure 1(d). Moreover, the expression of NPPA and NPPB was upregulated in H/R treated H9c2 cells (Figure 1(e, f)).

### Circ_0000080 knockdown attenuates hypoxia/reoxygenation-induced injuries in H9c2 cells

**Figure 2.**
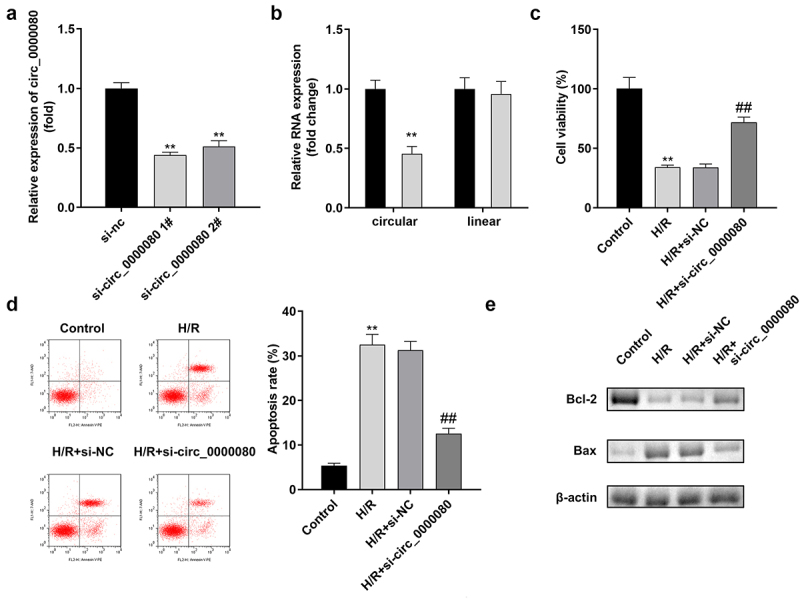
Knockdown of circ_0000080 inhibits H9c2 apoptosis induced by H/R. (a) The expression of circ_0000080 was downregulated in H9c2 cells by si-circ_0000080 1# (n = 3). (b) The circular characteristics of circ_0000080 was validated (n = 3). (c) Cell viability analysis with CCK-8 (n = 3). (d) Apoptosis analysis (n = 3). (e) Levels of Bcl-2 and Bax were examined by western blotting (n = 3). **p < 0.01, ##p < 0.01.

The expression level of circ_0000080 was markedly reduced by si-circ_0000080 1# compared to si-circ_0000080 2#, while si-circ_0000080 1# did not affect the linear GAPDH expression level (Figure 2(a, b)). Such finding suggests that circ_0000080 is indeed a circular transcript. As shown in Figure 2(c), h/R treatment markedly weakened H9c2 cell viability, which was remarkably improved by circ_0000080 knockdown. Hypoxia/reoxygenation-induced apoptotic cell ratio and Bax/Bcl-2 protein ratio were also substantially reduced by circ_0000080 knockdown in H/R-treated H9c2 cells (Figure 2(d, e)).

### Downregulation of circ_0000080 suppresses inflammation in H9c2 cells induced by H/R

**Figure 3.**
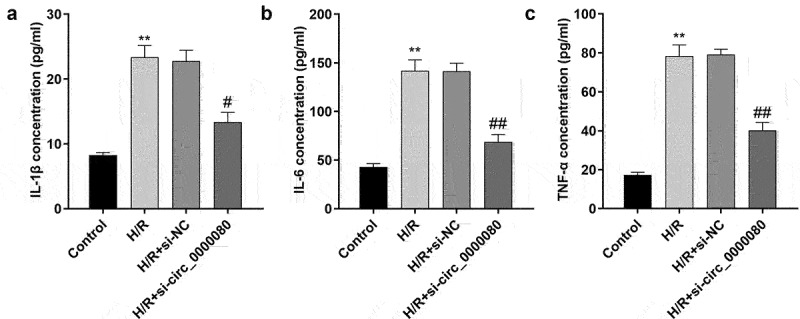
Knockdown of circ_0000080 suppresses H9c2 cell inflammation induced by H/R. (a) The concentration of IL-1β in H9c2 cells (n = 3). (b) The concentration of IL-6 in H9c2 cells (n = 3). (c) The concentration of TNF-α in H9c2 cells (n = 3). **p < 0.01, #p < 0.05, ##p < 0.01.

Inflammation after MI is necessary for heart repair; however, inflammation is also involved in the pathophysiological processes, such as heart remodeling and heart failure after MI [19]. As shown Figure 3, compared to control cells, H/R treatment/exposure significantly increased the release of IL-1β, IL-6, and TNF-α, which was reversed by knockdown of circ_0000080 (Figure 3(a-c)).

### Circ_0000080 targets miR-367-5p in cardiac myocytes

**Figure 4.**
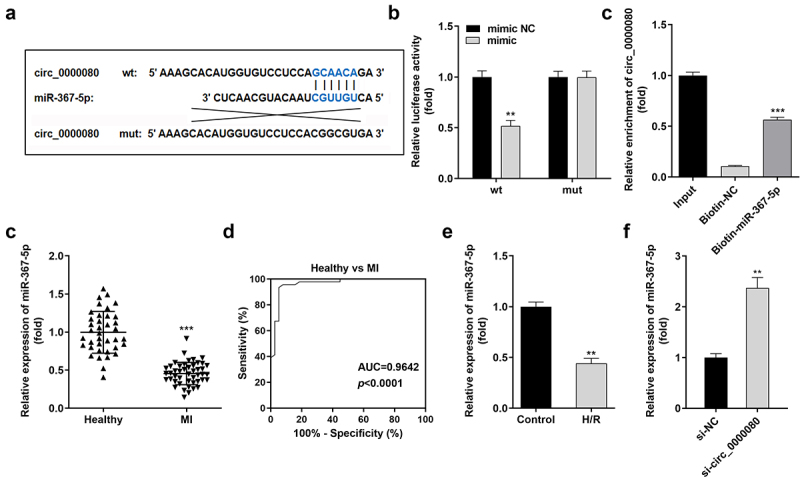
MiR-367-5p is a target of circ_0000080. (a) The binding site between circ_0000080 and miR-367-5p. (b) Luciferase activity of circ_0000080 3’‑UTR‑WT or MuT in H9c2 cells (n = 3). (c) RNA pull-down assay (n = 3). (d) MiR-367-5p was upregulated in patients with MI. (e) ROC analysis for the distinction between patients with MI and healthy controls. (f) The miR-367-5p level was analyzed by qRT-PCR (n = 3). (g) Detection of miR-367-5p expression in H/R cells (n = 3). **p < 0.01, ***p < 0.001.

Based on increasing evidence, circRNA acts as a miRNA sponge [20,21]. The binding sites of circ_0000080 and miR-367-5p are shown in Figure 4(a). MiR-367-5p mimics significantly downregulated the luciferase activity of circ_0000080 3’-UTR-wt in H9c2 cells (Figure 4(b)); however, no obvious changes were found in cells transfected with circ_0000080 3’-UTR-mut. The results of the RNA pull-down assay suggested that biotin-miR-367-5p could notably pull down more circ_0000080 (Figure 4(c)). Further, the expression level of miR-367-5p was significantly reduced in MI patients compared to normal controls (Figure 4(d)), and the corresponding AUC value of miR-367-5p for distinguishing between MI patients and healthy controls was 0.9642 (Figure 4(e)). Besides, the expression level of miR-367-5p was remarkably diminished in H/R-treated H9c2 cells (figure 4(f)). The qRT-PCR data revealed that the expression of miR-367-5p was reinforced in H9c2 cells with circ_0000080 knockdown (Figure 4(g)).

### MiR-367-5p knockdown reverses the effect of circ_0000080 knockdown in H/R-treated H9c2 cells

**Figure 5.**
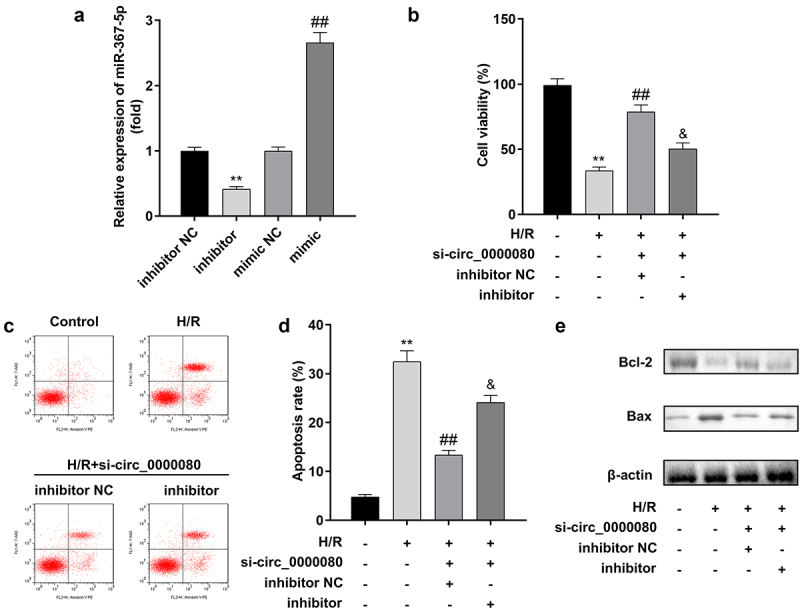
MiR-367-5p knockdown reverses the effect of circ_0000080 in H/R-treated H9c2 cells. (a) The expression of miR-367-5p (n = 3). (b) Cell viability (n = 3). (c-d) Flow cytometry (n = 3). (e) Western blot (n = 3). **p < 0.01, ##p < 0.01.

A rescue experiment was conducted to explore the relationship between circ_0000080 and miR-367-5p in H/R treated H9c2 cells. Based on qPCR, the miR-367-5p inhibitor significantly reduced the expression level of miR-367-5p, while the miR-367-5p mimic markedly increased the expression level of miR-367-5p (Figure 5(a)). The CCK-8 assay indicated that circ_0000080 knockdown reduced the viability of H/R-treated H9c2 cells, which was reversed by the miR-367-5p inhibitor (Figure 5(b)). Further, the miR-367-5p inhibitor alleviated the inhibition of H/R-treated H9c2 apoptosis induced by circ_0000080 knockdown (Figure 5(c-e)).

### Knockdown of miR-367-5p promotes inflammatory response

**Figure 6.**
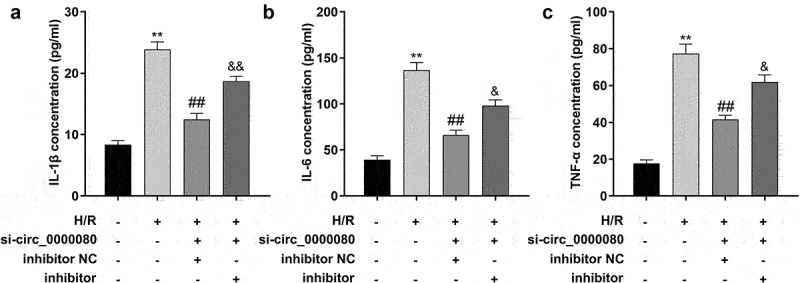
Knockdown of miR-367-5p reverses the reduction of inflammatory factors in H/R-treated H9c2 cells induced by circ_0000080 knockdown. (a) The concentration of IL-1β in H9c2 cells (n = 3). (b) The concentration of IL-6 in H9c2 cells (n = 3). (c) The concentration of TNF-α in H9c2 cells (n = 3). **p < 0.01, ##p < 0.01.

To determine the effects of miR-367-5p in H/R-treated H9c2 cells, an experiment was conducted to further assess the effects of miR-367-5p on the secretion of inflammatory factors in H9c2 cells in vitro. Knockdown of circ_0000080 reduced the release of inflammatory cytokines including IL-1β, IL-6, and TNF-α, and the miR-367-5p inhibitor was found to increase the secretion of IL-1β, IL-6, and TNF-α (Figure 6(a-c)).

### *MiR-367-5p directly targets* COX2 *in H9c2 cells*

**Figure 7.**
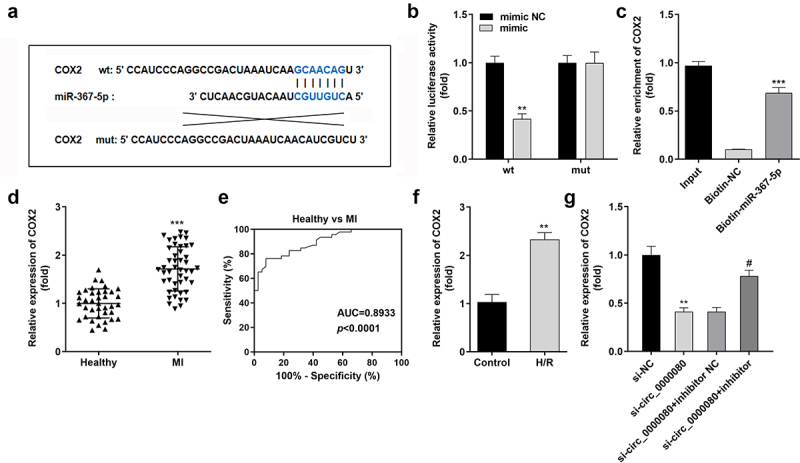
MiR-367-5p directly targets *COX2* in H9c2 cells. (a) The binding site between *COX2* and miR-367-5p. (b) Luciferase activity of *COX2* 3’‑UTR‑WT or MuT in H9c2 cells (n = 3). (c) RNA pull-down assay (n = 3). (d) *COX2* was upregulated in patients with MI. (e) ROC analysis for the distinction between patients with MI and healthy controls. (f) The *COX2* level in H/R-treated H9c2 cells was analyzed by qRT-PCR (n = 3). (g) The relationship among circ_0000080, miR-367-5p, and *COX2* (n = 3). **p < 0.01, ***p < 0.001, #p < 0.05.

The Starbase online software was used to predict the potential target of miR-367-5p to clarify the mechanism of the circ_0000080/miR-367-5p signaling pathway in MI. The binding sites of miR-367-5p and *COX2* are shown in Figure 7(a), and were further verified by the luciferase and RNA pull-down assays (Figure 7(b, c)). MiR-367-5p was found to inhibit the luciferase activity of the reporter carrying *COX2*-wt. Further, the RNA pull-down assay suggested that biotin-miR-367-5p could notably pull down more circ_0000080 (Figure 7(c)). The mRNA expression level of *COX2* was remarkably elevated in MI patients compared to normal controls (Figure 7(d)), and the corresponding AUC value of miR-367-5p for distinguishing between MI patients and healthy controls was 0.8933 (Figure 7(e)). Besides, *COX2* expression level was significantly elevated in H/R-treated H9c2 cells (figure 7(f)) but was reduced by circ_0000080 knockdown, with normal levels restored after transfection with the miR-367-5p inhibitor (Figure 7(g)).

### *Overexpression of* COX2 *reverses the effect of miR-367-5p in H/R-treated H9c2 cells*

**Figure 8.**
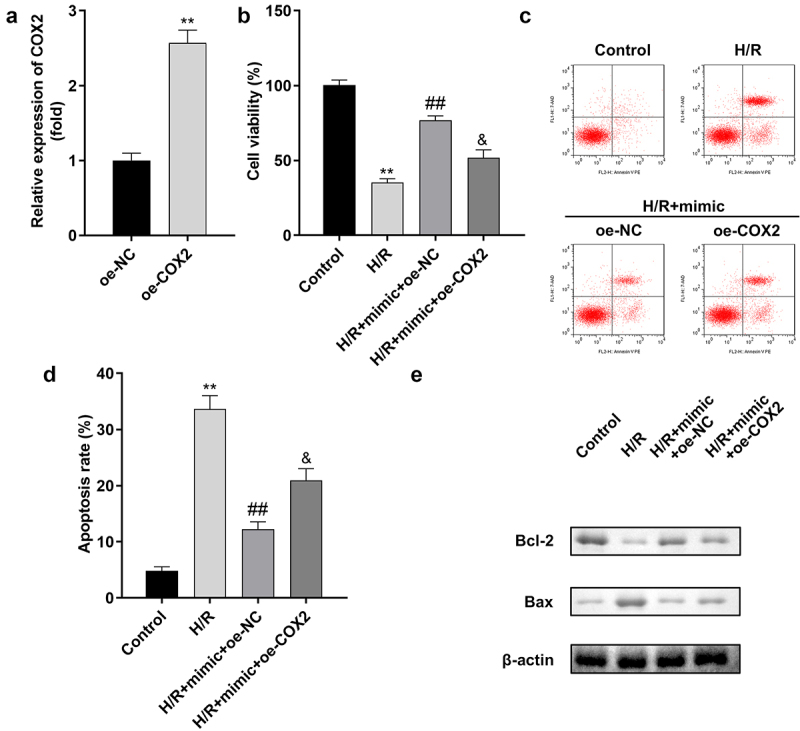
Overexpression of *COX2* reverses the effect of miR-367-5p in H/R-treated H9c2 cells. (a) The expression of *COX2* (n = 3). (b) Cell viability (n = 3). (c-d) Flow cytometry (n = 3). (e) Western blot (n = 3). **p < 0.01, ##p < 0.01.

A rescue experiment was performed to confirm the relationship between miR-367-5p and *COX2* in H/R-treated H9c2 cells. The transfection efficiency of *COX2* is shown in Figure 8(a). The viability of H/R-treated H9c2 cells was markedly improved by the miR-367-5p mimic, which was inhibited by *COX2* overexpression (Figure 8(b)). Moreover, *COX2* overexpression promoted apoptosis in H9c2 cells (Figure 8(c-e)).

### *Overexpression of* COX2 *reverses the reduction of inflammatory factors in H/R-treated H9c2 cells induced by the miR-367-5p mimic*

**Figure 9.**
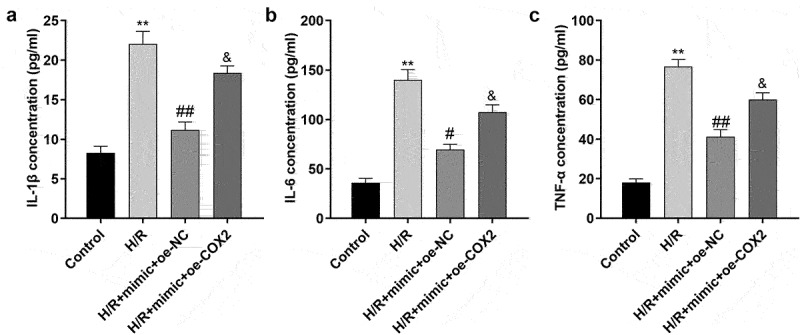
Overexpression of *COX2* reverses the reduction of inflammatory factors in H/R-treated H9c2 cells induced by the miR-367-5p mimic. (a) The concentration of IL-1β in H9c2 cells (n = 3). (b) The concentration of IL-6 in H9c2 cells (n = 3). (c) The concentration of TNF-α in H9c2 cells (n = 3). **p < 0.01, #p < 0.05, ##p < 0.01.

The overexpression of *COX2* significantly increased the release of inflammatory cytokines, such as IL-1β, IL-6, and TNF-α, as shown in Figure 9.

## Discussion

The cardiovascular system is the engine of life. Severe cardiovascular disease is a potentially fatal disease [22,23]. Although the standards for the prevention, diagnosis, and treatment of such diseases have significantly improved, the mortality rate associated with MI is still high [24]. Understanding the regulatory mechanism post-H/R injury is essential to improve the prognosis and treatment of MI. To our knowledge, this is the first study to reveal that circ_0000080 is upregulated in MI patients and in H/R-treated H9c2 cells. Moreover, the knockdown of circ_0000080 reduced the rate of apoptosis and inhibited inflammation induced by H/R. circ_0000080 targeted miR-367-5p in cardiomyocytes and miR-367-5p targeted *COX2*. MiR-367-5p knockdown or *COX2* overexpression inhibited the role of circ_0000080 in MI.

Apoptosis is a form of programmed cell death; it affects cardiomyocytes during MI [25]. The increase in the extent of hypoxia caused by blood flow interruption and the increase in the level of oxidative stress during ischemia-reperfusion can lead to myocardial apoptosis [26]. H/R-induced apoptosis and inflammatory response are two key factors in the progression of H/R-induced heart injury. Myocardial cells have low regenerative ability; thus, reducing their level of apoptosis and inhibiting inflammatory response in these cells can help protect a patient’s heart function and improve the prognosis [27]. CircRNAs play crucial roles in cardiac pathophysiological changes during heart failure. For example, the expression level of circ ACAP2 is upregulated in MI patients and it promotes apoptosis by binding to miR-29 [28]. Knockdown of circ_0060745 alleviates MI by inhibiting myocardial apoptosis and inflammation [29]. However, the regulatory role of circRNA in cardiomyocyte apoptosis is still unclear. Here, we studied the expression of circ_0000080 in MI patients and healthy controls – circ_0000080 was upregulated in MI patients and similarly upregulated in H/R treated H9c2 cells. To investigate the function of circ_0000080 in H9c2 cells, we sought to determine the effect of circ_0000080 on the viability of H9c2 cells. circ_0000080 knockdown was found to attenuate hypoxia/reoxygenation-induced injuries of H9c2 cells and suppress inflammation in cells. These results suggest that circ_0000080 inhibition can improve the function of cardiomyocytes by inhibiting apoptosis and decreasing the expression levels of inflammatory factors.

We proceeded to explore the mechanism of circ_0000080 in MI. Most circRNAs play regulatory roles in MI by targeting downstream genes [30,31]. Liu et al. found that circYAP1 binds to miR-367-5p and antagonizes the downregulation of p27 Kip1 by miR-367-5p, thereby inhibiting the proliferation and invasion of GC cells [32]. Accordingly, miR-367-5p was first predicted and identified as a novel downstream miRNA target of circ_0000080 in cardiomyocytes. In this study, miR-367-5p was found to be significantly downregulated in MI patients and H/R-treated H9c2 cells. Moreover, miR-367-5p knockdown reversed the effect of circ_0000080 and the reduction of inflammatory factors in cells. We revealed, for the first time, the function of miR-367-5p in MI and proved that miR-367-5p was targeted by circ_0000080 to regulate H/R damage.

To further study the mechanism of miR-367-5p’s biological function in MI, we investigated the downstream target gene of miR-367-5p. Zhang et al. found that *COX2* knockdown may increase the risk of MI [32]. Herein, miR-367-5p was found to directly target *COX2* in H9c2 cells, and the overexpression of *COX2* reversed the effect of miR-367-5p in cells. Therefore, circ_0000080 may weaken the targeted inhibitory effect of miR-367-5p on *COX2* by binding to miR-367-5p, thereby inhibiting the progression of MI. The circ_0000080/miR-367-5p/*COX2* axis may provide a new therapeutic option for MI.

The main limitation of this study is that the role of the circ_0000080/miR-367-5p/*COX2* axis in MI needs to be confirmed using several clinical studies, which will be the focus and a challenge of future research. Additionally, only the roles of miR-367-5p and *COX2* were explored in this study. whether other miRNAs and genes influence circ_0000080-mediated regulation in MI still needs to be elucidated.

## Conclusion

In summary, the circ_0000080/miR-367-5p/*COX2* signal axis was found to promote proliferation and impair the apoptosis of H/R treated cells. This study not only revealed the novel roles of the circ_0000080/miR-367-5p/*COX2* axis in MI, but also provided a new target for the management of MI.

## Supplementary Material

Supplemental Material

## Data Availability

The datasets used and analyzed during the current study are available from the corresponding author on reasonable request.
