# Supplementary material for: Role of circRNA circ_0000080 in myocardial hypoxia injury
Source: Bioengineered. 2022 Apr 27;13(4):10902–13. doi: 10.1080/21655979.2022.2066752 (PMC9208504; doi:10.1080/21655979.2022.2066752)
Supplement: Supplemental Material [file KBIE_A_2066752_SM6428.zip › supplementary/supplemented material 2 wild type and mutant sequences of circ_0000080.docx]

Wild type sequences of circ_0000080

Forward: 5’-AAGCTTAAAGCACATGGTGTCCTCCAGCAACAGAACTAGT-3’

Reverse: 5’-ACTAGTTCTGTTGCTGGAGGACACCATGTGCTTTAAGCTT-3’

Mutant sequences of circ_00000080

Forward: 5’-AAGCTTAAAGCACATGGTGTCCTCCACGGCGTGAACTAGT-3’

Reverse: 5’-ACATGTTCACGCCGTGGAGGACACCATGTGCTTTAAGCTT-3’
