# Supplementary figures and images for: Role of circRNA circ_0000080 in myocardial hypoxia injury
Source: Bioengineered. 2022 Apr 27;13(4):10902–13. doi: 10.1080/21655979.2022.2066752 (PMC9208504; doi:10.1080/21655979.2022.2066752)

Figure 2

Bax Bcl2 β-actin


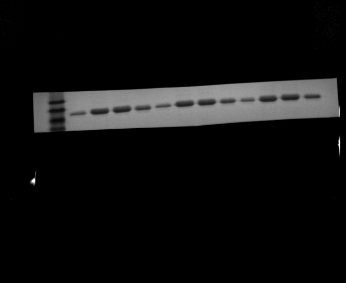

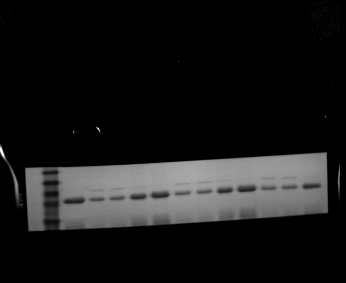

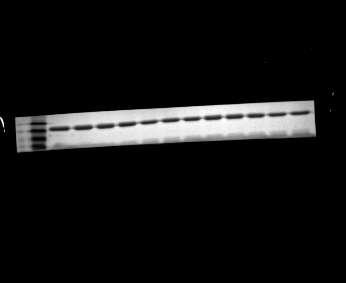


Figure 5

Bax Bcl2 β-actin


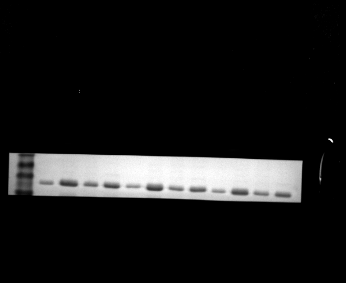

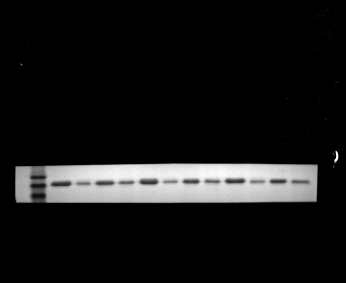

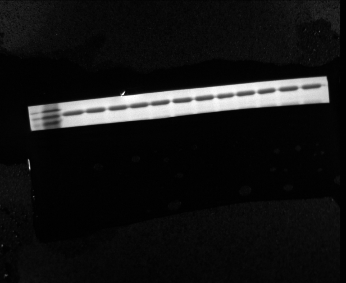


Figure 8

Bax Bcl2 β-actin


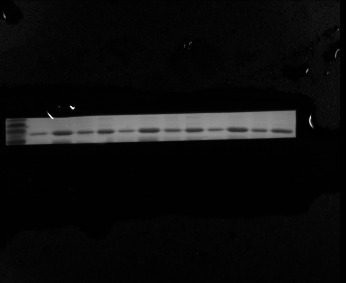

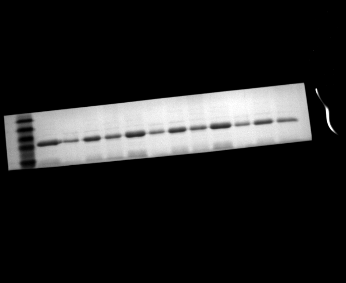

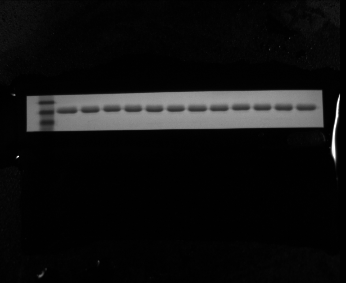

Supplement: Supplemental Material [file KBIE_A_2066752_SM6428.zip › supplementary/supplemented material 1 protein bands.docx]

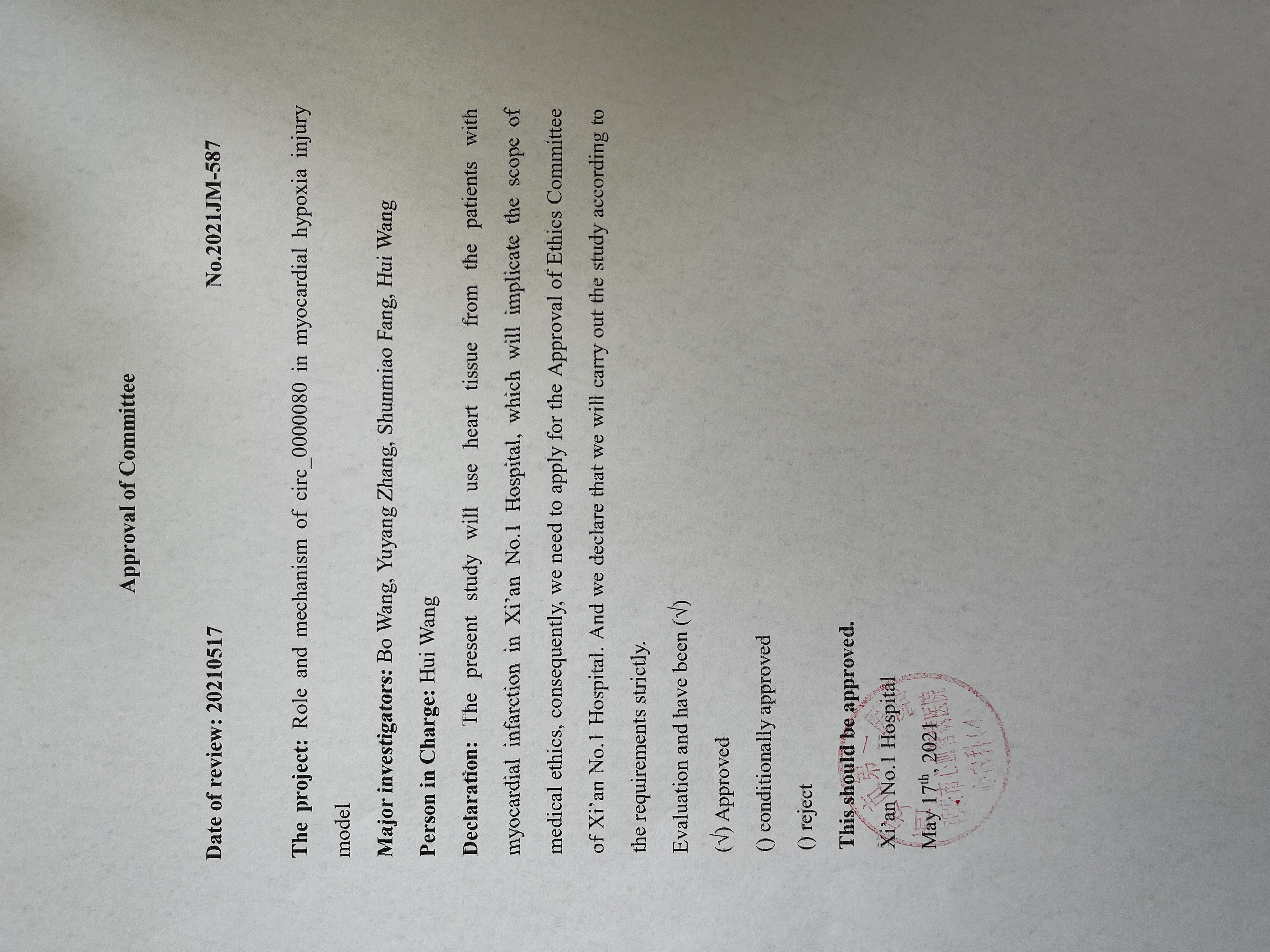

Supplement: Supplemental Material [file KBIE_A_2066752_SM6428.zip › supplementary/supplemented material 3 ethics approval.jpg]
